# An empirical study on the collaborative usability of age-appropriate smart home interface design

**DOI:** 10.3389/fpsyg.2023.1097834

**Published:** 2023-08-03

**Authors:** Chengmin Zhou, Wenjing Zhan, Ting Huang, Hanxiao Zhao, Jake Kaner

**Affiliations:** ^1^College of Furnishings and Industrial Design, Nanjing Forestry University, Nanjing, Jiangsu, China; ^2^Jiangsu Co-innovation Center of Efficient Processing and Utilization of Forest Resources, Nanjing Forestry University, Nanjing, Jiangsu, China; ^3^School of Art and Design, Nottingham Trent University, Nottingham, United Kingdom

**Keywords:** smart home, interactive interface, age-friendly design, optimal design, ease of use

## Abstract

**Introduction:**

The smart home has become a popular product, but with the development of the aging population, the differentiated characteristics of the elderly smart home products in terms of demand and use are becoming more and more significant. The existing smart products are complicated to operate and cumbersome to interact with, which increases the cognitive load of the elderly group and hinders the daily use and user experience feeling of the elderly. The purpose of this paper is to study the interface data information and interface visual design starting from hardware and software, interface interaction, to explore the better interface data information and interface visual design, and to output, a new prototype of the operating interface of smart home system for the elderly, so that the smart products can be better used by the elderly.

**Methods:**

Thirty-two participants aged 55–75 were invited to conduct the test, and subjective evaluation was conducted at the end of the test. Through the tests, the operability of the prototype structure for smart furniture systems for the elderly was demonstrated.

**Results:**

In terms of functionality a new task based on a combination of icons and text is proposed. In the control of devices, the switching status of devices, etc., needs to be clearly distinguished visually, eye-protective bright colors are used, paired with low saturation to highlight the focus, and high bright colors with gray to distinguish the device status. In terms of the density of the content, an appropriate proportion of images and text were used to make the information less dense. ln the arrangement of web content, information content relevant to users was placed first as much as possible.

**Discussion:**

Based on this, a secondary optimal design was carried out to improve the interactive design of the smart home for the elderly and output it as a prototype interactive interface. Thus, the operability, rationality, and aesthetic comfort of the prototype design of smart home interaction in an age-friendly scenario are improved, allowing the elderly to have a better experience when using the smart home.

## 1. Introduction

With the rapid growth and popularity of the Internet of Things (IoT), the term “intelligence” has become an umbrella term for innovative technologies with a degree of artificial intelligence as the technology and environment continue to evolve. An important feature of smart technology is to perceive and respond accordingly to external information acquisition (Chan et al., 2008; Balta-Ozkan et al., 2014). Since the long-term purpose of smart technology is to benefit humanity, it has become the backbone of innovative ideas such as the “smart home” (Alam et al., 2011; Dawid et al., 2017; Han et al., 2022). The shift in products and services has led to an increase in the operability of devices, which has driven an increase in the number of smart home technology transactions worldwide (Khedekar et al., 2017). As a result, the smart home is becoming a popular product, that is, the use of IoT technologies in the daily life of the home. It plays an important role in providing users with convenience, efficiency, and quality of life (Marikyan et al., 2019). Smart homes can control and monitor environmental changes in people's homes (Pan et al., 2022), determine user behavior, detect anomalies (Fahad and Tahir, 2021), and contribute to better family-to-family control (Bissoli et al., 2019). In addition, the study showed a significant correlation between the use of smart home services and wellbeing (Sesto et al., 2012). Many studies have demonstrated the benefits of smart homes, but the current research revolves around the technical characteristics of smart homes, i.e., the lack of a user perspective, especially in the elderly user group (Marikyan et al., 2019).

The increasing pressure of survival in modern society, the expanding proportion of older people, and the gradual increase in the age of childbirth have made the problem of aging increasingly serious. Globally, the population of older adults is growing rapidly, and it is estimated that by 2050, the number of older adults over 60 will double from 12 to 22% of the population, with 80% of these older adults expected to live in low- and middle-income countries (Yu et al., 2022a). In this context, the physical, emotional, behavioral, and cognitive aspects of older adults will undergo significant changes, and it is particularly important to explore the real needs of older adults (Yang et al., 2018). As older adults have more free time, their physical and psychological problems are magnified in this context (Xiong et al., 2018). In terms of physiology, it has been shown that more than half of the elderly over 65 have visual impairment, one third have hearing impairment, and two fifths have motor impairment, and that their physical condition deteriorates with age (Nebeker et al., 2021). This means that care design for the elderly population needs to take full account of their physical functioning problems, taking into account their visual and hearing difficulties (Chang and Östlund, 2019; Lindström and Ståhl, 2019). In response to the psychological and physical needs of the elderly, assistive devices or caregivers are needed to take care of their daily lives and spiritual-based humanistic care (Andrews et al., 2019). However, most elderly people, cannot afford long-term in-home care. The emergence of smart homes is a great way to do this (Fox and Connolly, 2018). Smart homes can not only make life easier and safer for the elderly but also provide a sustainable solution for long-term in-home care (Rashidi and Mihailidis, 2013; Sharma and Wong, 2020). Smart homes can help older adults live independently, especially those who suffer from motor control disorders (Chen et al., 2022). Past studies have shown that older adults are receptive to life changes and benefits such as emergency assistance, fall prevention and screening, and medication alerts from smart home devices (Crespo et al., 2006; Chen et al., 2013; Hu and Ning, 2016). Therefore, the design of smart homes is considered a key factor in improving the quality of life and protecting the health and safety of the elderly.

The supply of smart homes and other healthy senior products suitable for the older population is increasing in demand as health needs grow (Lee et al., 2006). However, studies addressing the current state of communication among older adults have found lower usage of smart devices (e.g., cell phones) compared to younger adults due to uncontrollable factors such as the decline in perceptual and cognitive abilities over time. Smart devices continue to be upgraded, and communication and communication when used by older adults has become a significant problem (Majumder et al., 2017; Eiguren Munitis et al., 2021). Lack of experience in independent use and difficulties in product operation have become major barriers for older adults to use products (Axisa et al., 2005; Kim et al., 2010). With the increase of smart home functions and complexity, the communication and control of smart devices become more and more inconvenient for the elderly, who are unfamiliar with digital technology and whose physiological and cognitive learning levels are constantly in decline as they age. They experience difficulties in correctly operating digital systems not only because of their vision and physical strength, but also because they forget the operation process and steps due to their intelligence and memory, as well as lack the ability to communicate and understand (Kim et al., 2010; Cho and Kim, 2014; Po-Chan, 2019; Cheng et al., 2022). Studies show that more than 50% of seniors encounter problems related to product design every day in their daily lives. Only 53% of them are well-trained to use quality products for their own needs (Wang et al., 2020). Therefore, to effectively address these issues, attention and focus on smart home product design are needed, and along with technological advances, it is crucial to develop user-friendly device interfaces. How the elderly can be more simple and easy to understand in the interaction process of operating smart homes has become an important research topic, and being able to truly allow the elderly to use and enjoy smart homes has become a key goal in solving this problem. In this regard, although smart homes can provide a certain convenience for the elderly, the user experience and interaction of the elderly in this process is an issue worthy of attention (Yang et al., 2016). In the field of smart home, with the continuous development of home automation, Internet of Things and other technologies, users will interact with smart home more and more. Therefore, from the interaction mode, the user experience design concept is applied to the interactive operation interface design of smart home to benefit the elderly.

Current solutions for the age-appropriate use of smart homes focus on research related to the use of sensors in smart solutions, research related to the implementation of remote access functions in smart home systems to monitor the elderly and operate devices through mobile devices rather than fixed devices, smart home operational benefits and smart scenario output and whether smart home design solutions have the potential for direct application and the cost of smart home value. Scholars' research has focused on studies on user-centered factors of smart home affordability (Cho and Choi, 2020) or personalized user interface frameworks based on Eclipse Smart Home and Universal Remote Console to enhance the user experience when using smart homes (Smirek et al., 2016) among others. In the controlled experiments with the older and younger groups, objective experimental data and subjective ratings then showed that: (1) Elderly preferred figurative icons compared to the younger group (Backhaus et al., 2018; Chen et al., 2020). (2) The younger group had better user performance for button sizes of 15 mm and above, while the older group had better performance for button sizes around 20 mm. (3) Participants in the younger group had better user performance for graphics vs. text ratios of 1:1 and 1:3, while the user performance of the older group participants was better at 1:3 (Yu et al., 2022b). In terms of user experience, existing research demonstrates that augmented reality and affective computing at the edge enable social robots to be better companions for older adults (Anjum et al., 2021). The emergence of smart environments and social robots proves that environment-based smart homes can provide integrated care services suitable for the elderly (Anghel et al., 2020). The current research results of ICT (Information and Communications Technology) with intelligence, personalization, and adaptivity as the core can actively, healthily, and effectively respond to aging and improve the operability of smart home interfaces (Giakoumis et al., 2019). In summary, most of the current international research on a smart home is centered on smart home-related technologies, which are very extensive and have high depth and relevance, while China's research in this field is still in its initial stage. Especially for smart homes for the elderly, related research is still weak, and domestic smart home research is still mostly focused on technological innovation of smart home systems, lacking analysis of user preferences and ease of use of operating interfaces (Colle and Hiszem, 2004; Cheng et al., 2019). Few studies have also used systematic methods and evaluation metrics to study the interaction and subjective experiences of older adults in using smart homes. Touch screen control, video control, and voice control are the three most common human-computer interaction methods in smart homes, and because the declining physical functions of the elderly have an impact on human-computer interaction, mobile application interfaces need to be designed to match the behavioral abilities of the elderly. The touch interface, on the other hand, allows natural and convenient human-computer interaction (Geva et al., 2013; Fahad and Tahir, 2021), making it easier and more convenient for inexperienced users (Marikyan et al., 2019), and therefore, it was chosen as the display carrier for smart homes in this study. In the touch screen, the comfortable and easy-to-understand operation interface helps to develop a user-centered smart home and is more likely to propose solutions to the problems of lack of independent use and difficulties in product operation among the elderly (Mittelstädt et al., 2014).

Therefore, the focus of this paper is on the study of human-computer interaction interfaces in smart homes for the elderly-adapted population, especially touch-screen interfaces aimed at supporting the usability and user experience of the elderly. The research in this paper is as follows: (1) Taking the factors of the self-care elderly, spatial scene, interaction behavior, behavior object, and experience demand in the smart home age-friendly scenario as the research basis, the smart scene panel is used as the design carrier of the age-friendly smart home interaction system, and the prototype design of the age-friendly smart home interaction interface is carried out with the themes of convenience, simplicity, and warmth, while two special functions, i.e., scene opening and scene presetting. This can reduce unnecessary operations for the elderly when setting up devices in different scenes, which usually do not exist in smart home interactive systems. The prototype contains the functions of scene opening and closing with one click, personalization, operation guidance, warm reminder, and touch voice. (2) In terms of details of operational data and information, functional buttons are important components for users to interact with smart home devices, and the size, style, and operation mode of functional buttons may affect user experience. This paper identifies four operational problems in the operation prototype and optimizes them through experiments, and studies several operations such as new tasks, information modules, and control devices. (3) This study also pays much attention to the subjective preferences of the elderly in terms of visual interface aesthetic performance. In terms of interface aesthetics, firstly, the findings of the prototype design study by Demiris and Hensel (2008), Zhou et al. (2022a), and Zhou et al. (2022b) were combined with the questionnaire. The prototype interface was designed to verify the conclusion that the 18 mm functional buttons, up and down sliding method and simple style in the interactive interface of the age-friendly smart home are better subjectively evaluated through satisfaction testing, followed by the design points such as interface theme color, key color setting points, information density layout and content priority on the page. The aesthetic design of the interface can be more in line with the aesthetics of the elderly and enhance their sense of user experience. (4) Through the deepening test of the experiment, the original interface was re-optimized and experimented again to ensure that the output intelligent interface system can be better facilitated for the elderly to use, studied, and optimized from both functional and visual perspectives, solved the pain points of the elderly, reduced the cognitive and usage burden of the elderly group, enhanced its ease of use, and output a set of interaction prototypes. On the one hand, this study facilitates the life of the elderly and strengthens digital technology communication with the outside world; on the other hand, it provides smart home services for the elderly, which can reduce the consumption of human resources and ease the burden of aging on families and society; it helps elderly users improve their independence in life and enhance their psychological self-recognition and live happier. Finally, the research on the interactive prototype of smart home systems deepens the development and direction of interface design and user experience. In addition to the construction and enhancement of technology, economy, and ecology, this paper provides new ideas to improve the user experience of a smart home.

## 2. User requirements elements disassembled and built

### 2.1. Demand disassembly

This user study of age-friendly smart homes was conducted using a questionnaire distributed to a healthy and self-sufficient elderly group between the ages of 55 and 75, all with normal communication comprehension and textual understanding. It contained demographic questions (age, gender, income, education level, etc.), habit and opinion questions, specifically divided into single sections on smart home usage, user physiology, lifestyle habits, and basic user profile. A total of 155 were collected. The Ethics Committee of the Science and Technology Division of Nanjing Forestry University approved the study protocol (Jiangsu Province, China), which was read and signed by all participants before participation.

In the questionnaire, the respondents were asked to select the level of importance of different aspects of the smart home interaction system according to their needs, which were classified into five levels: very unimportant, unimportant, indifferent, important, and very important (Park et al., 2020). This survey was an attitude scale, and the quantitative information collected in the questionnaire was examined, especially the reliability and accuracy of the attitude scale questions were studied with the help of reliability analysis. The Cronbach reliability analysis of the Smart Home Interactive Function and Experience Needs Scale is shown in Table 1, and it can be found that the value of the reliability alpha coefficient is 0.777, which is greater than 0.7, indicating that the quality of the reliability of the research data is good. According to the column of “alpha coefficient of deleted items”, we can see that there is no increase in reliability after deleting any item, so each item can be used and should not be deleted.

**Table 1:** Cronbach's reliability analysis of the smart home interaction function and experience requirements scale.

**Item**	**Correction term total**	**The alpha**	**Cronbach**
	**correlation(CITC) of**	**coefficient of**	**alpha**
	**the deleted item**	**the deleted item**	**coefficient**
Beautiful operation interface	0.493	0.753	
Large text and prominent	0.448	0.760	
Simple operation and few steps	0.555	0.753	
Clear information	0.551	0.753	0.777
All functions are included in the	0.456	0.753	
Personalized interface	0.493	0.467	
Personalized features	0.493	0.538	

Through the reliability analysis, the design requirement points with excellent reliability were retained for screening.

The validity of the design of this question was checked using validity analysis: with a KMO value of 0.829 and a KMO value greater than 0.8, the study data were well-suited to extract information and responded with good validity. The *p*-value was met by Bartlett's test. However, it can be seen from Table 2 below: for the common degree, a total of 3 items involving prominent information, comprehensive function, and personalized customization interface, their corresponding common degree values are less than 0.4, which indicates that the information of the research items cannot be expressed effectively. Therefore, after deleting these 3 items and analyzing them, the validity analysis of the smart home interaction function and experience requirements scale is shown in Table 2.

**Table 2:** Validity analysis of the smart home interaction function and experience requirements scale.

**Object**	**Factor loading factor**	**Commonality (common factor variance)**
Beautiful operation interface	0.649	0.421
Large text and prominent information	0.598	0.358
Simple operation and few steps	0.706	0.498
Clear information	0.698	0.488
All functions are included in the interface	0.609	0.371
Personalized interface	0.628	0.394
Personalized features	0.694	0.482
KMO value	0.829	-
*P*-value	0.000	-

The design requirement points with excellent validity were retained for screening by validity analysis, *p* < 0.05.

The 5 levels of very unimportant, unimportant, indifferent, important, and very important are scored, respectively −2, −1, 0, 1, and 2. According to the average score, users think that the aesthetics of the operation interface is the most important when they use the interface, followed by clear and easy-to-operate information, and the importance score of the remaining items is between important and indifferent. To summarize the interactive experience needs and priority order of smart home: beautiful operation interface >clear information not easy to operate wrongly >simple operation with few steps. Smart home interaction function and experience demand scale scores are shown in Table 3. Finally, the questionnaire also investigates the user's life troubles and speculates the smart home functions that users may need. The survey results show that the most prominent distress of users is vision loss, and 100% of the elderly group over 65 years old chose this item. Therefore, we should focus on the visual design of the interface when designing the interactive interface of the age-friendly home, which needs to be simple and clear, with not too many elements and not too small text and icon sizes. Therefore, in the subsequent development of the interface, we chose a 1:3 graphic ratio, with black font and white background. At the same time, we noticed that elderly people prefer to read figurative icons rather than text, and considering the overall aesthetics of the interface, we set the font size to 18 mm to highlight the icon display. In addition, nearly one in seven users chose the item of poor memory, so the functions of schedule reminders and medication reminders should be considered in the development of a smart home operating system.

**Table 3:** Smart home interaction function and experience demand scale scores.

**Item**	**Score**	**Average score**
Beautiful operation interface	168	1.135
Clear information, not easy to operate error	79	0.534
Simple operation and few steps	61	0.412
Personalized features	6	0.041

The analysis found that users consider interface aesthetics to be the most important, followed by clear information and easy operation, and finally personalization and customization.

**Figure 1:**
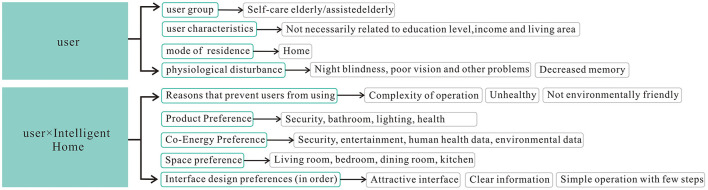
Summary of questionnaire research results. The different colors in this figure indicate the different layers of the hierarchy. The button with blue background is the first level, indicating a summary from the user and the user with the smart home; the basket box button is the second level; and the button with white background is the third level. It serves as the main basis for the subsequent user goal disassembly.

After the quantitative user study of the questionnaire research, a qualitative study of user interviews was conducted to understand how the age-friendly smart home should adapt to users' living habits and how users would use the product when, where, and for what reasons, aided by the user observation method. A total of six users, all aged 60 and above, were screened through the questionnaire as suitable for further user interviews. The interviewees had experience in using smart home products and services, as well as those who had no experience in using smart home but had experience in using other smart devices and were potential users who were willing to try using smart home products or services. The interviews started from three types of oriented questions, such as goal, system and process, and recorded the users' rating rate, problem, feeling and expectation of using smart home. The results show that users will generally go out every day and need to see the time and weather conditions before going out, and most users have a relatively regular life. In terms of equipment, most users use smart home function is relatively single, and many smart products are eventually used as ordinary products, the reason is still that the interaction experience is poor, and the operation process is complicated and will not operate. Second, most users believe that the linkage between devices is insufficient. In terms of user preferences, typing and search functions are generally difficult or time-consuming for users, who prefer light-colored interfaces and do not like interfaces with too many information content elements. Therefore, in the subsequent interface development, light color is adopted as the theme color of the page, the overall style is simple, the home page needs to reflect the time, weather, and other situations in a large area, and the operation simplifies the process as much as possible, and develops one-key open and close functions according to different scenarios. User research was conducted through questionnaire research and user interviews to obtain user characteristics, lifestyles, and needs from a macro perspective (Joshi et al., 2015), which guided the interface design and operational functions of the subsequent system development. The specific results are shown in Figure 1.

### 2.2. User objective disassembly

The three levels of cognitive and affective processing correspond to the three types of user goals, namely experience goals, final goals and life goals (Al-Maskari and Sanderson, 2011). User goals are a part of the main manifestation of the persona model, and user goals drive users to produce different behaviors.For example, users open the window every morning to ventilate and get fresh air, and the reason for ventilating is derived from the pursuit of healthy life. Based on the questionnaire and user interview results discussed above, two types of user models are constructed, namely, A users who pursue simple and convenient life and B users who pursue a beautiful interface and high-quality life.

Class A users who pursue simplicity and convenience are the entry-level experience of smart home for aging. To ensure that the system is easy to learn and to reduce the cognitive load and learning cost of the system, it is necessary to have a strong readability of the interface content, smooth interaction process, simple operation, clear functional instructions, reasonable layout of interface elements, uniform interaction form, so that users are willing to accept and learn, and have a good emotional experience (Han et al., 2022).

B users who go for a beautiful interface and high quality of life have more experience in using smart products, have higher requirements for smart home interaction experience, need to set and control devices according to preferences, have more diversified requirements for interaction forms, need to be reasonably concise in functions, clear interaction logic, strengthen the scene mode multi-device linkage, and have deeper requirements for interface beauty and layout (Pan et al., 2022).

**Figure 2:**
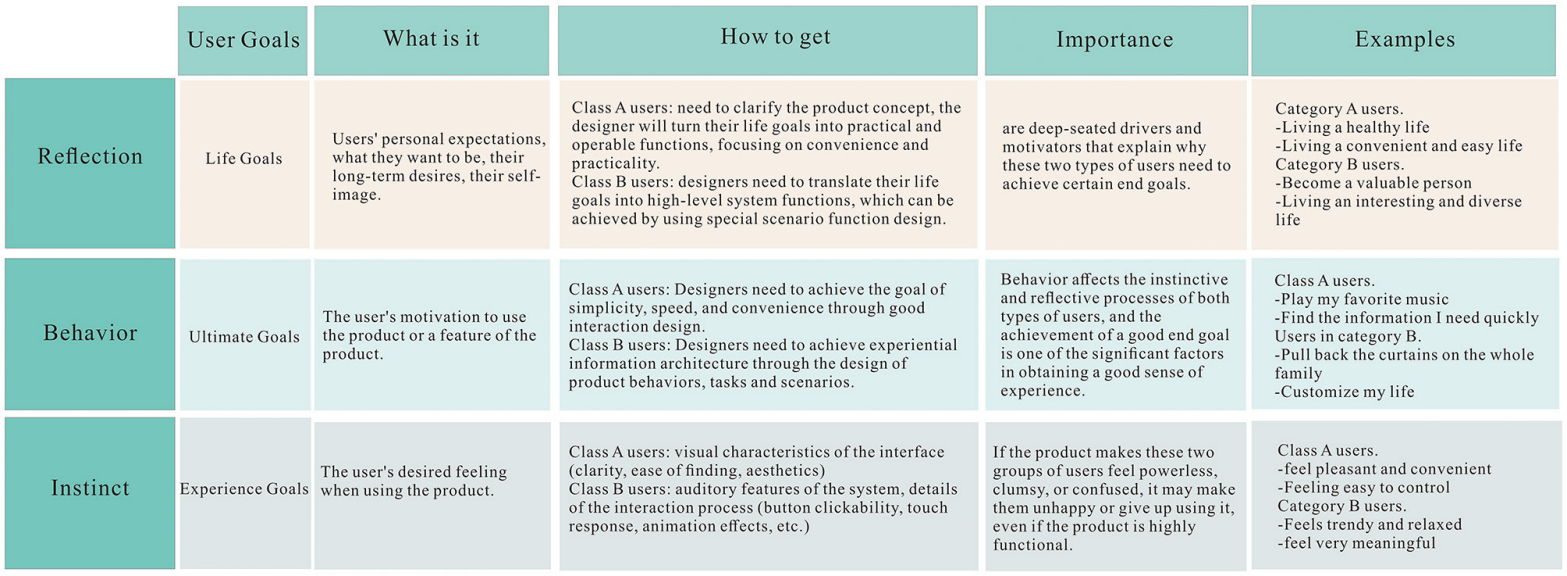
Target analysis of target users. This figure illustrates the three levels of cognitive and affective processing in product design proposed by Donald Norman for each of the two different types of users mentioned above, starting from instinct, behavior, and reflection, and analyzing them in different aspects.

The goal analysis of the two categories of target users constructed in this paper then draws on the three levels of cognitive and emotional processing in product design proposed by Donald Norman: instinct, behavior, and reflection (Rose and Levinson, 2004). The goal disassembly is shown in Figure 2. The goal disassembly of the two types of target users guides the subsequent prototyping, requiring us to focus on more advanced features and interface aesthetics based on satisfying the needs of the former type of users. Therefore, our prototype design starts from both functional and formal aspects to ensure the beautiful and simple interface and smooth and fluent operation. Strengthen the important aspects and weaken the unimportant details. At the same time, we also needed to increase the variety of interaction forms. Therefore, multi-device linkage, voice interaction and other functions were added to the development.

Through quantitative and qualitative analysis, the user pain points were derived and the user needs were refined, and the goals of two different types of users were dismantled, thus summarizing the design direction of this age-friendly smart home system as follows: (1) Interface and visual level. The interface needs to be clear and easy to understand while maintaining aesthetics. In the development, we chose a 1:3 graphic ratio, an 18 mm button size, a light color as the theme color of the page, a black content expression on a white background, and an overall simple style. To clarify the interface design details need to conduct experiments to further explore the interface factors that meet the user's operating habits. (2) System level. Relying on scene mode and room mode to achieve smart home multi-device linkage, the user experience of using smart home is enhanced and the difficulty of using it is reduced by reducing the user's operation steps on the devices in the fixed scenes of daily life. (3) Interaction mode level. The main interaction mode is touch, which is simpler than other interaction modes and can give elderly users enough space to think and react; voice interaction is used as an auxiliary to touch so that users can use voice interaction when it is inconvenient to touch or when they need to operate more conveniently and quickly. (4) Functional requirements. Easy access to daily information, warm prompts according to the scene, operation guidance, clear meaning of icons, one-click control of multiple devices in the scene mode, detection of the environment, and feedback data.

### 2.3. Description of user interaction behavior

The scenarios of users using smart homes in different situations are classified. To explore the development of the scenario mode function of controlling multiple devices with one click, the interaction task flow structure words required for user requirements are expressed with the help of a flowchart, and the user's operation history is visualized with the help of the preliminary design of the prototype page, and the specific scenarios are designed as follows.

Under the scenario of starting scenario mode, users can switch to the scenario panel by clicking the item button in the bottom sidebar, turn on or off the scenario by clicking the corresponding scenario in the scenario panel, double-click or long-press the scenario to enter the editing page of the scenario module, ordinary users can turn on the scenario with one click in the “Scenario” page, and users with more needs can personalize the scenario by clicking the home button in the bottom sidebar. Users with more needs can personalize the scenario, and click the Home button in the bottom sidebar to go back to the home page.

In the case of a room or a device that you want to turn on in a room, you can click the “Room” button in the middle of the upper area of the page to enter the room page, where you can turn on or off all the devices in a room with one click. If you want to view the details of the room or view the equipment in the room and make settings for the equipment, you can double-click or long-press the room to see the details of the room, click the equipment on this page to turn it on or off, double-click or long-press the equipment to make adjustments.

**Figure 3:**
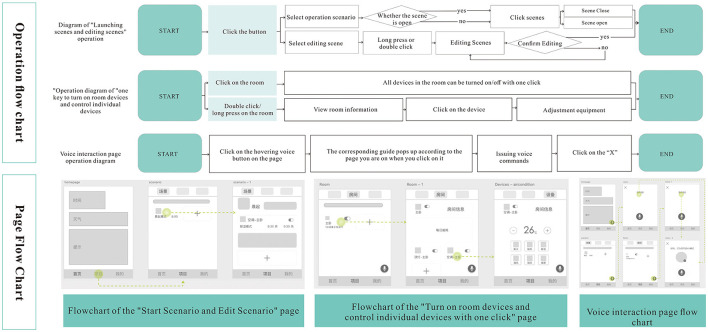
Operation flow chart and page flow chart. It includes scenarios such as starting scenes, editing scenes, opening room devices and regulating individual devices with one click, and voice interaction.

Under the scenario of voice interaction, the user can enter the page of voice interaction by clicking the voice button hovering at the bottom right corner of the page, and depending on the page where the user is before clicking the voice button, the guidance words on the pop-up voice page will be different. When the user turns on voice interaction on the home page or the scene page, the instruction will be biased to instruct the user how to give voice instruction to turn on a scene mode, how to record a schedule, how to play music review, etc. When the user turns on voice interaction on the room and device pages, the instructions will guide the user on how to issue voice commands to search and find a room or device or how to schedule a single device. The operation flow and page flow under the scenarios of starting scenarios, turning on devices, and voice interaction, as shown in Figure 3.

### 2.4. Interface prototype construction

Through the research and analysis of user needs and objectives and the construction of activity scenarios, combined with the results of the prototype design study conducted by Zhou et al. (2022b). It was concluded that the design points of this prototype for its age-appropriateness were as follows: (1) A simple style with lines was adopted, and the information was conveyed in black characters on a white background. (2) The size of the graphic ratio was 1:3, and 18 mm was chosen for the functional buttons. (3) In the layout features of the homepage, a simple and intuitive multi-column layout or card layout is used, combined with a bottom navigation bar type of main navigation. (4) In the selection of the main color, a light monochrome with low saturation is used as the main color to highlight key information with a saturated tone (Lindberg and Näsänen, 2003; Norman et al., 2003; Smith, 2009). Its age-appropriateness was continuously confirmed and optimized in subsequent experiments, and finally, a set of operational interaction fidelity prototypes were produced as follows.

1) First-level page

**Figure 4:**
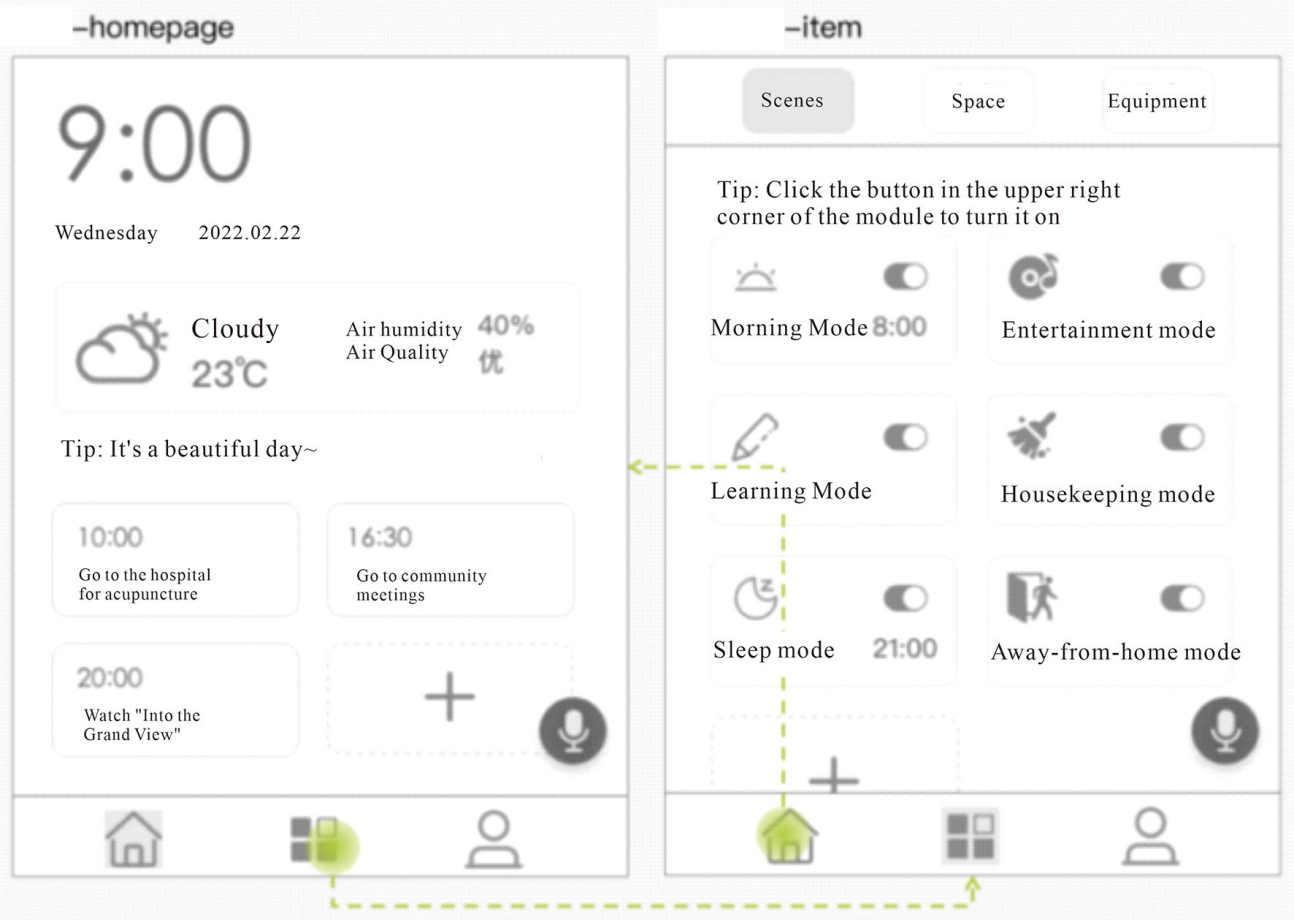
Age-friendly smart home panel level 1 page. Including home panel and project panel.

The first-level page is mainly the home page and the project panel, as shown in Figure 4. The home page is a daily information display page made to match the age-friendly scenario, mainly with a time module, environment module, and warm reminder module. The time module is a dynamic time, showing the year, month, day of the week, and time. The environment module focuses on weather information, outdoor temperature, air humidity, and air quality information display. The time module is one of the indispensable daily information that aging users need to get, and it takes up a large space in the home page for the problem of declining eyesight of the elderly. Click the second button “Project” in the bottom bar to enter the smart home function opening mode, and recommend users to use the scene mode. Under the “Scene” selection on the project page, users can perform the preset scene mode one-click opening operation, and the switch in the upper right corner of each preset scene module shows the running status of the scene. At the same time, for the sake of aesthetic user needs for personalization, double-click or long press the scene module to enter the corresponding scene editing page.

2) Scene opening and setting page

**Figure 5:**
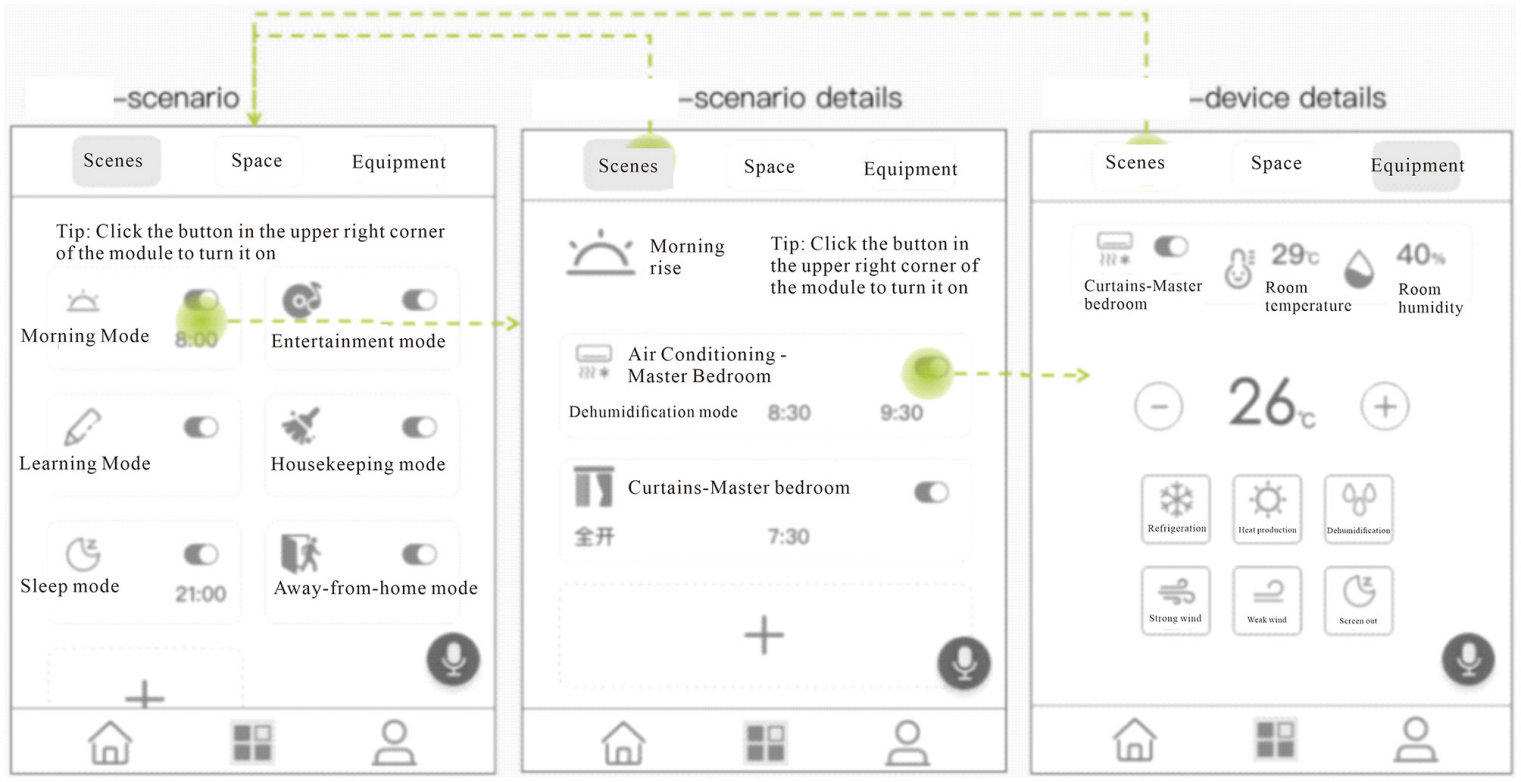
Age-friendly smart home panel scene mode related pages. Including scene page, scene detail page, and device detail page.

There are three main pages involving scenes, namely the scene page, the scene details page, and the device details page. Ordinary users, they can turn on or off the scene with one click on the scene page. Users with more advanced needs can add more device behaviors to the mode on the scene details page, or turn on or off the preset device behaviors, as in Figure 5.

3) Rooms and settings page

**Figure 6:**
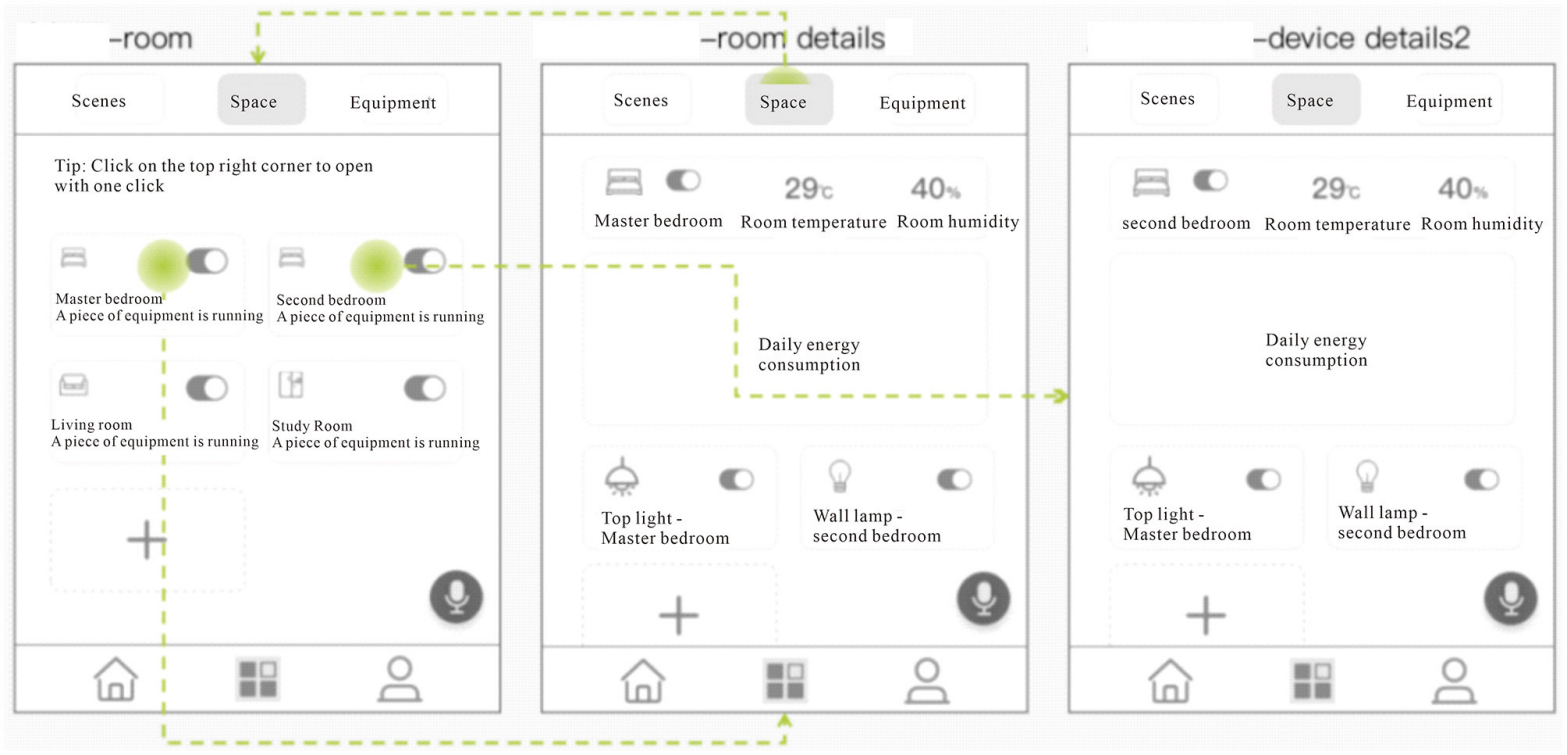
Age-friendly smart home panel room and settings page. The picture shows the one-touch to turn on the control devices in the room.

In addition to the scene module also designed for the user room module, the user can turn on the equipment in the room with one click, or through the room details page to view the room information, energy consumption, the user can also use the room module to view what devices are in the room and operate the device, the design logic is the user usually turn on a device will go to the room where the device to operate the device or to find its remote control behavior habits, such subconscious. The design logic is that users usually go to the room where a device is located to operate it or to find its remote control. This kind of subconscious design helps the user to perceive and smooth the operation process, as shown in Figure 6.

4) Voice and voice feedback page

The hearing and memory of elderly decrease with age, and single-channel speech interaction may increase the cognitive burden of users. In voice interaction, speech is the language-based means of interaction, aided by visualization of visual information, thus improving the interaction experience of voice users and improving the multichannel perception of voice interaction for the elderly (Rose and Levinson, 2004). By adding voice interaction, the opening of scenes, control of devices, and access to information can be completed conveniently and quickly.

**Figure 7:**
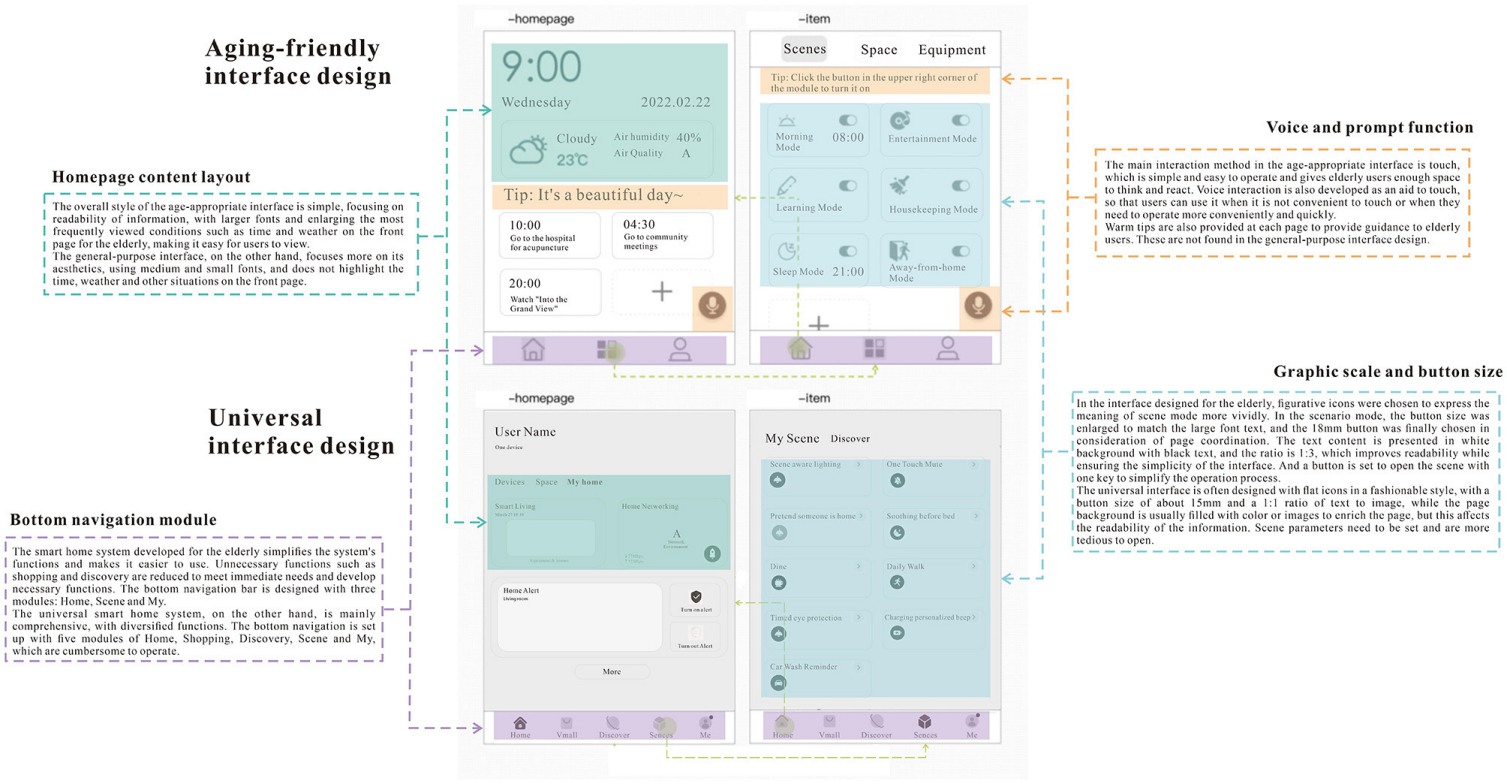
Comparison of the age-friendly prototype and the generic prototype. The H brand was compared with the “Home” and “Scenes” sections of the interface design. The top is the age-friendly prototype designed for this study, and the bottom is the generic prototype of H brand. The green part is the comparison of the content layout of the home page, the purple part is the comparison of the bottom navigation module, the blue part is the comparison of text and icons, and the yellow part is the comparison of voice and prompt functions.

Finally, to verify the suitability of this interface design for the elderly, the interface of the general-purpose smart home system of H brand (China) was first extracted, and the two interfaces of “Home” and “Scenes” were processed with low fidelity, and then compared with the interface of this design. The four aspects of the home page content layout, bottom navigation module, voice, and prompt function, and graphic ratio and button size all prove that the design direction of the age-friendly smart home system summarized above is well-responded to in this interface design, and it is more suitable for the elderly compared with the general-purpose interface. The analysis process is shown in Figure 7.

## 3. Interface prototype testing

### 3.1. Experiment and materials

#### 3.1.1. Experimental stimulus materials

Axure (Axure Software Solution, San Diego, CA, USA) and MockingBot (Beijing Modaokeshi Technology Co., Ltd., Beijing, China) were used for the prototype of this experiment. as the support for the interface design. To check the usability and satisfaction of the prototype, four different operation tasks were set, namely “View daily information board”, “Scene opening board”, and “Voice interaction board”. “Voice interaction board” and “Device control board”, the difficulty varies, and need to operate separately.

#### 3.1.2. Experimental equipment

The experiments were conducted in a controlled environment, controlled illumination, and a sound reduction chamber. The framework of the experiment was deployed on a 10.5 in iPad air with a 12-megapixel rear wide-angle camera and horizontal stereo dual speakers. Participants were invited to sit on an adjustable chair in front of the flat screen. Participants' operation time and a number of jams were recorded and analyzed, and during the operation test, which operated the predetermined four tasks, subjects were asked to ensure a minimum distance of approximately 60 cm from the display during the experiment. The height of the webcam and the respondents' eye height were kept at the same level to ensure that the experiment would not have a large time error due to the tricky angle. A questionnaire on the aesthetic assessment of the interface was also filled out upon completion.

#### 3.1.3. Experimental subjects

This experiment was conducted in the laboratory of the School of Home and Industrial Design at Nanjing Forestry University. A total of 32 healthy, independent elderly people, aged 55–75, were recruited, 17 of whom were female (159–173 cm) and 15 male (166–180 cm). To ensure the accuracy and validity of the sample, the 32 subjects were asked to be free of eye swelling, eyelid ptosis, eye disease, or eye surgery within 6 months, and to wear glasses with natural or corrected visual acuity over 1.0 for myopia or presbyopia; in addition, subjects were asked to be free of physical or cognitive impairment, with the usual hand being the right hand. All study subjects were retired teachers or residents living on campus.

The ethics committee of the Science and Technology Division of Nanjing Forestry University approved the study protocol (Jiangsu Province, China). All subjects will read and sign the trial before participating in the trial and will be compensated for the trial upon completion.

#### 3.1.4. Experimental procedure

(1) Experimental purpose

The reason for conducting prototype testing is to discover the functional and experiential problems of the age-friendly smart home interactive prototype, evaluate its age-appropriateness, and verify the suitability of the designed prototype for designing an age-friendly smart home system. The test scenarios are constructed by using storyboarding to create scenarios for the subjects and make them complete the test tasks to obtain more realistic user experience feedback.

(2) Functional testing

Construct test scenarios against already designed interaction prototypes. When building test scenarios, virtual scenarios built using interactable prototypes, described in the form of stories or scenarios, are used to test tasks in key activity scenarios of users. The specific functional test scenarios contain four main types of tasks as follows.

1) In the view daily information section, task one is to get information such as time, weather, and outdoor environment. It is suitable for going out grocery shopping, going out, and other scenarios. Through the smart panel to check whether it is suitable to go out, whether it is necessary to add clothes, etc., you need to ask the user to check the panel and say the result.

2) In the scene opening section, task two finds the scene page and opens the scene with one click. It is suitable for scenes such as buying groceries and starting to cook, listening to music while cooking after turning on cooking mode, etc. Users are required to find scenes and turn them on.

3) In the voice interaction panel, task three is to find the voice button and issue a command to open the leisure mode, after dinner in the living room watching TV, using the smart panel to issue a voice command to open the leisure mode scene, the user needs to use the voice function to open the leisure mode.

4) In the device control panel, task four is to find the device list and the master bedroom air conditioner and adjust the temperature of the master bedroom air conditioner. Suitable for scenarios such as hot weather and adjusting the air conditioning temperature of the master bedroom alone, users can also use the voice function to find them. The experiment was conducted for 15-20 min, with a 5 min break after completing the first two tasks to ensure the subjects' eye comfort, and the subjects were randomly selected and completed the tasks as required, and the entire experiment was recorded.

(3) Interface evaluation

After the interface use test, the subjects were asked to complete a questionnaire to evaluate the interface design of the prototype, to study this chapter on the age-ability of the intelligent home interaction design interface, to verify the validity of the research methods and strategies in this study for realizing the interaction interface visually responding to the ageability scenario, to discover the problems in the prototype design and to make improvements from the interface design. The Likert Scale was used to rate the satisfaction level of the interactive interface of the age-friendly smart home system, and the satisfaction was classified and scored, and the evaluation was divided into very satisfied (5 points), satisfied (4 points), average (3 points), dissatisfied (2 points), and very dissatisfied (1 point). The evaluation results were obtained by counting the satisfaction rating questionnaires filled by the subjects and calculating the mean value to further deepen the aesthetics of the prototype.

### 3.2. Data statistics and analysis

#### 3.2.1. Analysis of operational data

**Figure 8:**
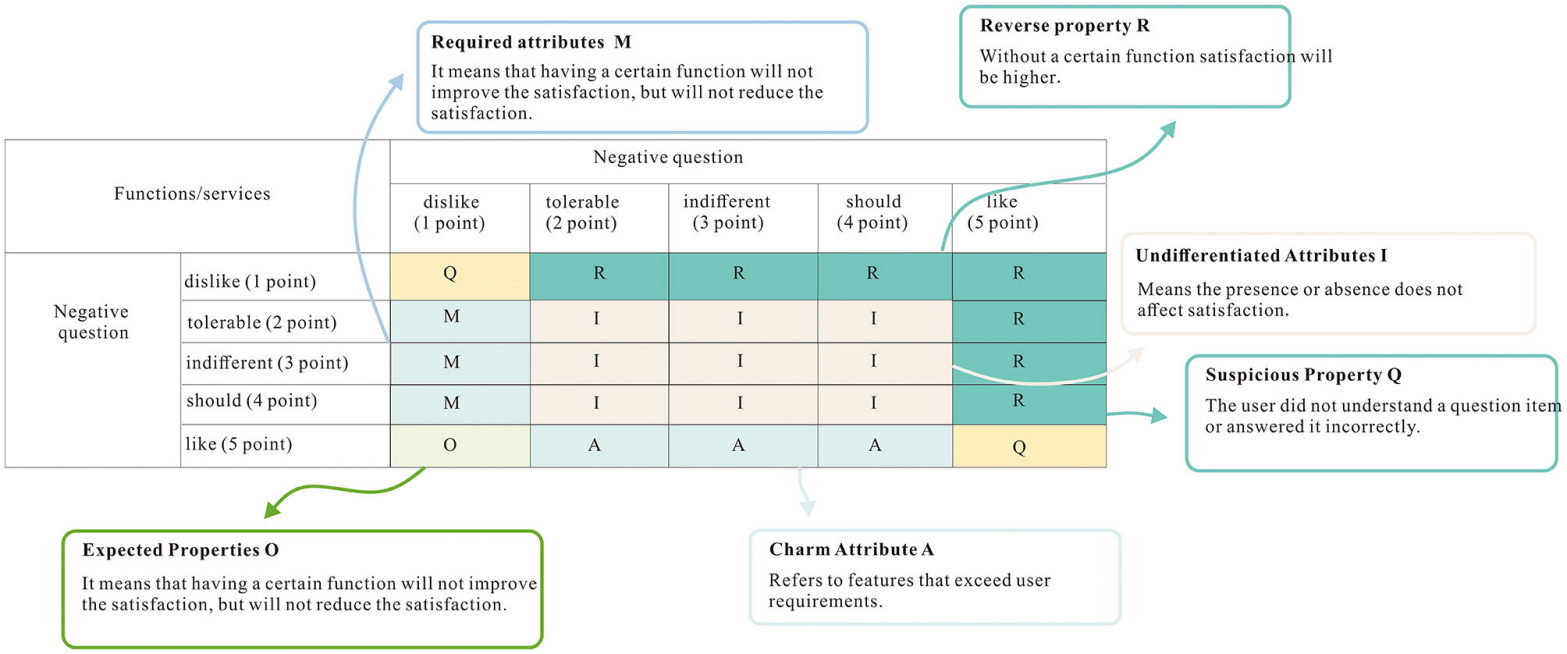
Cross-tabulation of Kano model evaluation results. The same function was asked separately from both positive and negative questions, and then the cross-tabulations between the options of positive and negative questions were summarized.

Using the Kano model to study the relationship situation between functional requirements and satisfaction, the same function was first asked from both positive and negative questions, and then the cross summaries between the options of positive and negative questions were obtained for a total of 6 attributes. The cross-tabulation of the Kano model evaluation results is shown in Figure 8.

As shown in Table 4, F represents a function, A represents a charming attribute, O represents desired attribute, M represents an essential attribute, I represents an undifferentiated attribute, R represents a reverse attribute, Q represents a suspicious attribute, and CR represents classification result B represents better W represents worse. The functions are 1 for daily information as a display, 2 for warm tips, 3 for operation guidance, 4 for scene presetting, 5 for voice feedback, and 6 for voice touch combination (Sharif Ullah and Tamaki, 2011). After the analysis of the results, it can be seen that in the 6 functions according a certain attribute accounted for the most as the division boundary, and finally in the 6 functions with the necessary attributes there is one, for the daily information display. There are two charming attributes, namely, warm tips and scene preset. There is one desired attribute, which is operation guidance. The combination of voice feedback and voice touch is an undifferentiated attribute. This result can better reflect the previous research and design direction and is analyzed as follows.

**Table 4:** Charisma attribute study. Charisma attribute analysis was performed for 6 functions.

**F**	**A (%)**	**O (%)**	**M (%)**	**I (%)**	**R (%)**	**O (%)**	**CR**	**B (%)**	**W (%)**
1	0.00	25.00	41.67	33.33	0.00	0.00	M	25.00	−66.67
2	41.67	25.00	8.33	25.00	0.00	0.00	A	66.67	−33.33
3	16.67	58.33	16.67	8.33	0.00	0.00	O	75.00	−75.00
4	50.00	25.00	0.00	25.00	0.00	0.00	A	75.00	−25.00
5	0.00	0.00	41.67	58.33	0.00	0.00	I	0.00	−41.67
6	8.33	8.33	41.67	41.67	0.00	0.00	I	16.67	−50.00

(1) Because the elderly have difficulties in giving specific and standardized voice commands, voice interaction is used as an auxiliary means of interaction when defining product features. Voice interaction as an auxiliary function does not have a great impact on satisfaction, but according to the charm attribute study, its undifferentiated attributes and essential attributes have the same score, so the combination of voice interaction and touch control of the two control methods will not improve user satisfaction, but without voice, interaction may be a slight decline in user experience.

(2) The analysis results show that “Daily Information Display” is a necessary attribute, time and weather are the information users need to get in their daily life, daily information display is not a special feature and will not significantly improve user satisfaction, but the absence of this feature will affect the user experience.

(3) The “Warm Reminder” and “Scene Preset” functions are charming attributes, without which user satisfaction will not drop significantly, which can explain why there are few such functions in the existing domestic smart home systems. However, for the elderly user group, the scene preset can simplify the operation steps and improve efficiency, which is in line with the research results in Chapter 3. The warm reminder function addresses the pain point of memory loss of the elderly and brings emotional care to the elderly, thus enhancing user satisfaction.

(4) “Operation Guidance” is the desired attribute, without which user satisfaction decreases and with which user satisfaction increases. The analysis of the questionnaire results shows that without this feature, user satisfaction decreases because users need to think about how to do the operation or make trial and error to complete the task during the operation.

In summary, the order of importance of the function development of the age-friendly smart home should be “Daily Information Display” >“Operation Guidance” >“Scene Preset” >“Warm Tips” >“Voice Touch Combined Interaction”, which is basically in line with the previous research results and can provide a reference for development priority in the subsequent system iteration and upgrade process.

#### 3.2.2. Visual data analysis

The content of the questionnaire is about the interface design, including the overall look and feel, layout and icon style and size, text size satisfaction issues. Specifically, it includes: Whether, the overall appearance of the interface is good. Whether, the layout of the interface elements is reasonable. Whether, the size of the text in the interface makes you feel clear and readable. Whether, the size of the icons in the interface makes you feel satisfied. Whether, the sparsity of the interface layout makes you feel satisfied. Whether, the style of the icons in the interface makes you feel satisfied, etc.

The evaluation results were obtained by counting the mean values of the satisfaction rating questionnaires filled by the subjects. The overall mean score of the interface design of the age-friendly smart home interactive system is 5.53, and the users are most satisfied with the text size and icon size, which are 6.5 and 6.08, respectively. It means that users are satisfied with the interface design in general, the icon size is the highest, and the satisfaction with the interface layout is the lowest, only 4.42, which needs to be improved. Users were asked in detail about the layout of the interface and found that the reason for the low score was that they thought the main function “Scene Preset” should be placed on the home page or in a position that is easier to find, and that the current design did not allow them to complete their tasks faster.

**Figure 9:**
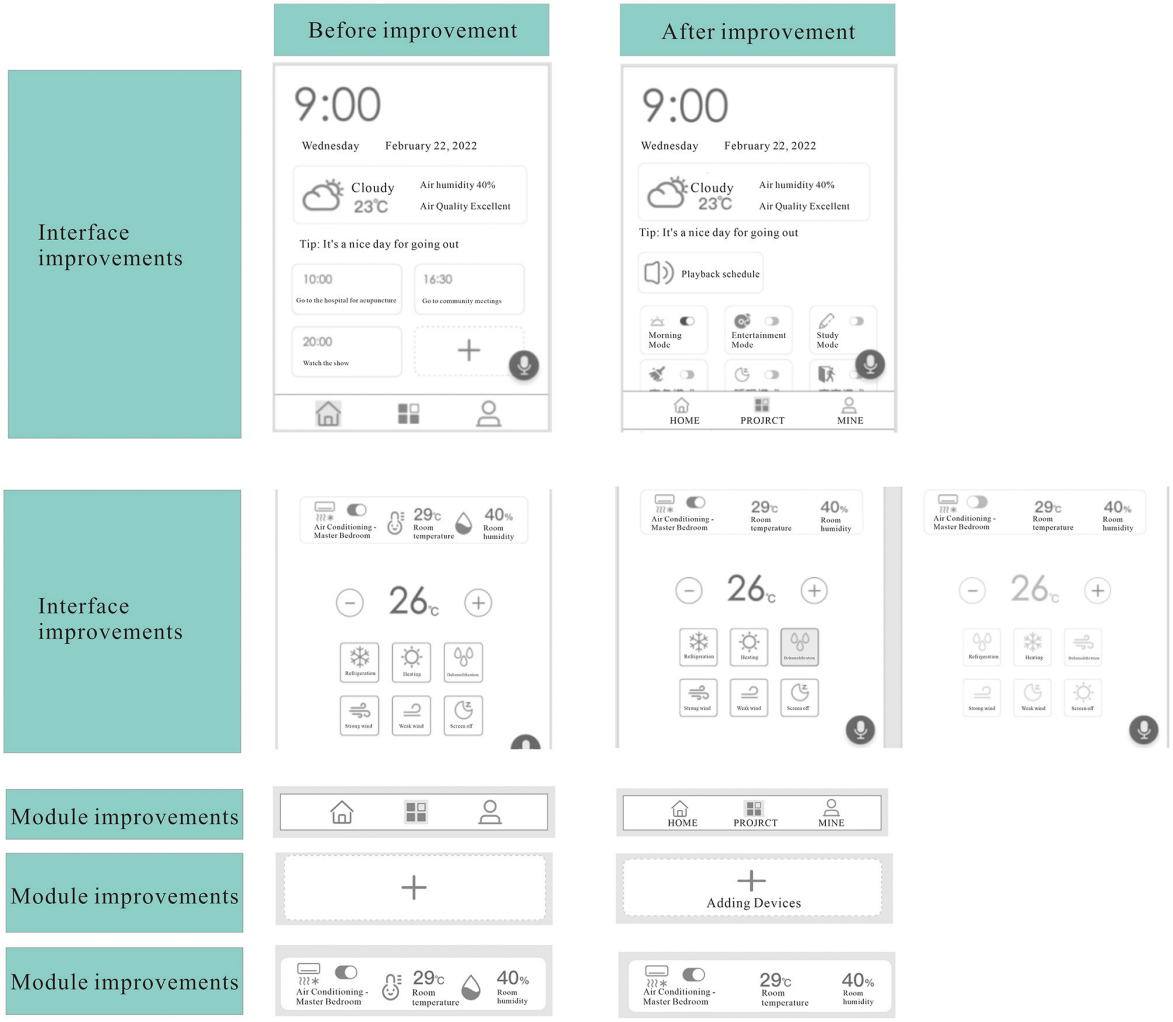
Operation scheme improvement. Before and after the improvement of the interface is listed, the optimization of the icon meaning, new task, device control, information density.

After the test is completed, the data is organized and analyzed. (1) In the view schedule information task, users do not understand the meaning of the plus sign in the schedule area, and the icon for a new schedule is changed to “+” and the text “New Schedule”. In the scene opening task, the meaning of the icon in the bottom sidebar is not indicated, so users cannot understand it accurately. (2) Users think the step of clicking the “Project” button in the middle of the bottom sidebar and then opening the scene is not simple enough. We need to add text labels to the icons in the bottom sidebar, adjust the scene mode to the home page, and adjust the display position of the schedule. (3) In the device control task, there are too many elements in the device information column, which increases the user's cognitive cost and the uncertainty of whether the device is turned on or not on the device control page. It is necessary to change the main information in the information module from the upper part to the lower part and concentrate it in the hot zone of the thumb, to reduce the crowding of elements and enhance the convenience of operation, as well as to distinguish the page status when the device is switched on or off. (4) In the device control task, it is not sure whether the device is on or not on the device control page, and it is necessary to add a page state that distinguishes when the device is switched on or off. The improvement scheme is shown in Figure 9.

The first improvement is the interface improvement. Before the improvement, the schedule reminder was placed in the lower part of the home page, after the improvement, the main function “Scene Preset One-click On” was adjusted to the lower part of the home page and concentrated in the thumb hot zone, reducing the interaction steps for users to use the scene function.

The second improvement is the interface improvement for the device control page. Before the improvement, the switch status of the device is not obvious, but after the improvement, the low saturated gray color is used to imply that it cannot be clicked and the visual color difference with the main color green is used to convey the signal of refusing to click this button. The gray device control icon is used to indicate that the device is turned off on the current page, while the green color indicates that the device is clickable or not yet turned on. The color and the on/off button function are used to indicate the on/off status of the device.

The third improvement to the module is the improvement to the bottom navigation bar. Before the improvement, the three buttons in the navigation bar were not marked, which made it difficult for users to understand the meaning of the buttons, after the improvement, the 3 buttons are marked with text, which makes the meaning more clear and reduces the cognitive load of users.

The fourth improvement is the improvement of the module, which is the improvement of the design of the new or added button. Before the improvement, the button only had a plus icon, and the user could not understand its meaning at first, but the improvement is the combination of the plus icon and the text mark to help the user understand and find the function.

The fifth improvement is the improvement of the module, which is the improvement of the device information column. Before the improvement, there were too many elements, which affected the efficiency of the user's information acquisition, after the improvement, the necessary information was left to improve the user's cognitive efficiency.

### 3.3. Visual optimization

The visual optimization stage mainly includes the specification design of visual elements of the age-friendly smart home interaction interface and the high-fidelity design of the interaction interface. The experiment proved through subjective evaluation that users accept and like the 18 mm function buttons, the up and down sliding method, and the minimalist style in the existing conclusion. It was also found that different usage scenarios required the application of different colors. The theme color is green, as green usually contains the semantics of “Pass” and “Success”, and is also very eye-friendly and senior-friendly, used to indicate “Task Success”, “The Device is Running”, etc. and to highlight and guide older people to click. The color of the icons is divided into two categories: green indicates that the corresponding function button is highlighted, indicating that the scene, room, or device is on or running, and the green bottom bar icon indicates that the current page is under that category. The gray icon indicates that the scene, room or device is not yet on. In terms of the information density of the page arrangement, a moderate proportion of graphics is used, and the information density is low. In terms of the arrangement of the page content, priority needs to be given to the information content with high user relevance. In terms of the information density of the page arrangement, a moderate proportion of the text is used with a low information density to facilitate the view of the elderly group. In the arrangement of the page content, information content with high relevance should be highlighted as the focus.

**Figure 10:**
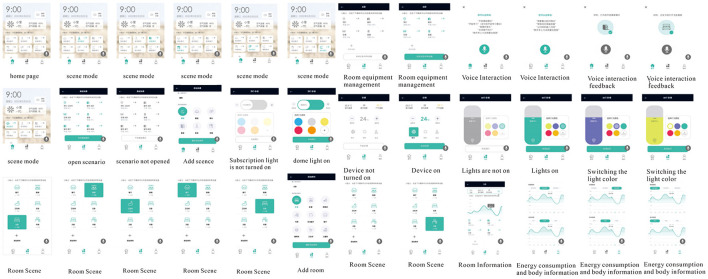
Output the final high-fidelity prototype design interface. Used 1:3 graphics in page layout, larger text ratio, 18 mm icon size, and color split between green and gray. The information density is low, and the information content with high user relevance is amplified. The function contains three sections: Home, Scene, and My, and developed special functions such as one-key opening scene and voice interaction.

Finally, the functional and visual problems found in the prototype test were optimized and landed, and the design scheme was deepened and improved, which finally improved the interaction design practice of the age-friendly smart home system and output the prototype of the high-fidelity interaction interface of the age-friendly smart home system. Finally, according to the experimental results, the problems of functionality and visibility that appeared in the prototype verification were optimized, and the design was deepened and improved, to realize the human-computer interaction design of the smart home for the elderly and form a set of human-computer interaction interface with high fidelity. The design of color and font specification, buttons, icons, and interactable modules are shown in Figure 10. Finally, invited 32 subjects (all of whom had participated in Experiment 1) to test the optimized output interaction interface, and the testers were satisfied with the overall satisfaction of this optimized design, as well as with the optimized five operation optimization and visual optimization.

## 4. Discussion

Smart homes are rapidly gaining popularity and more and more consumers are using smart home systems. To make more people accept and use smart homes, especially the elderly, it is necessary to make the interface of smart home simple and easy to use. This study focuses on the pain points of the elderly group, which can effectively help designers identify the design features of smart home interfaces in terms of form and function, to better improve the ease of use and achieve a better user experience. A combination of literature research, quantitative analysis of questionnaires, and qualitative analysis of interviews was used to identify users' needs and goals and refine the focus in specific usage scenarios. The experiments in this study aim to optimize the prototype of the interaction interface designed in the first round and to examine whether the use of the prototype is easy and beautiful for users in terms of both operational experiments and visual evaluation. The interface design was optimized in terms of color, font size, icon size, etc. and the final design results were output. The interface design was optimized through satisfaction experiments, such as reducing the interaction steps of the user using the scenario function and developing a one-click opening function. The buttons were switched between low-saturated gray and highlighted green to signal whether the device was on or not. The text was added to the bottom navigation bar buttons, and the labeling used a combination of icons plus text to create new tasks. When arranging the web content, informative content with a high degree of relevance to the user was placed first whenever possible, etc. Finally, the existing findings of the research on button design, module design, and icon design in the age-friendly smart home interaction interface were also verified.

### 4.1. Operating functions

The existing smart products are complicated to operate and cumbersome to interact with, which increases the cognitive and usage burden of the elderly and hinders their daily use and user experience. This paper designs an interface prototype of an age-friendly smart home system based on previous research results and questionnaire analysis, and experimental testing proves that the overall usage process of the age-friendly smart home prototype designed in this paper is smooth and consistent with previous research results. However, there are shortcomings in the operation details, such as users do not understand the meaning of the plus sign in the schedule area in the task of viewing schedule information, users think that the step of opening scenes by clicking the “Project” button in the middle of the bottom bar is too complicated, and there are too many elements in the device information bar, etc. Further improvements can be made through experimental analysis to include the main function. The main function of “One Click to Open Scene Preset” is adjusted to the lower part of the home page to reduce the interaction steps for users to use the scene function, use color and switch button function to indicate the switching status of the device, add text labels for the 3 buttons to make the meaning clearer and reduce the cognitive load of users, use plus icon and text labels to help users understand and find, etc.

### 4.2. Interface form

Interface design is increasingly focused on the subjective preferences of users, but this is largely ignored in smart interface design guidelines. User selection of products is largely based on subjective preferences. Therefore, it is necessary to focus on user perceptions and preferences. The subjective questionnaire analysis proved the existing findings of the studies on button design, module design, and icon design in age-appropriate smart home interaction interfaces noted in the previous studies. In terms of interface aesthetics, the interface was optimized and improved in details, such as using green as the theme color to indicate “Task Successful”, “Device Running”, etc. and to highlight the icons to guide the elderly to click on them, and the icons can be divided into two categories according to the color: highlighted In terms of the information density of the page layout, it adopts a moderate proportion of graphics and text with low information density. Tn the arrangement of the page content, it gives priority to the information content with high relevance to users. It not only improves the operability of the prototype design of smart home interaction in the age-friendly scenario, but also improves the beauty and comfort of the prototype design, so that the elderly can have a better experience when using the smart home.

### 4.3. Limitations

In this study, only middle-aged and elderly people with good vision were selected as experimental subjects. It is not clear whether different postures and usage environments will have an effect on the users' behavior compared to those related to elderly vision, eye diseases, and sensory disorders. When users manipulate the smart home device interface, they are not necessarily in a sitting position. They are likely to stand, lie down, or walk, and the effects of the above factors on the experimental results need to be further explored. Second, the volume of the voice control interface can affect the test results, especially when the subjects heard voice commands to perform the task. In subjects with good hearing, they will move faster and easier, and vice versa. The third reason is that the task in the experiment was relatively homogeneous, allowing the subjects to complete the task easily and satisfactorily. However, in reality, when users wish to operate in the smart home device interface, they are expected to complete a series of searches and constant clicks. This difference will have an impact on the results of the study. Fourth, the test method and the final analysis of the experiment were mainly derived from the subjective evaluation of the subjects, and the process did not combine with the subjective evaluation, although some objective data such as the number of blockages and the frequency of clicks were recorded, which is the deficiency and shortcoming of this experiment at this stage, and is the direction and research topic to continue our experiment subsequently. Furthermore, the illustrations included in this research proposal are limited to conceptual diagrams and images related to the study product, which we will expand on in subsequent studies. We will also focus more on disability in the target group, which is often the case, and this aspect of the study is meaningful and important and points to our future research goals. Finally, although the timeout of using smart home products in the home may cause certain safety hazards, it can be more harmful to the elderly group of users if it is not operated properly. This study combines safety, ease of use, and satisfaction with subjective assessments to examine user behavior. Elderly will respond more slowly; therefore, the validity of the task completion time indicator should be explored in depth. Future work should focus on a combined subjective and objective evaluation system, further research on system construction and technology, and a multifaceted evaluation system. Although some of the above factors are reflected in this paper, there is still much to explore, and we hope that future research will focus on these aspects.

## 5. Conclusion

In this paper, we developed a design framework to improve the usability of smart home systems for ageing and exported an interaction prototype. A user study test with 32 participants verified the key points of interaction prototype ease of use design and optimization: operational flow test and interface evaluation test. The accuracy of set target values, task completion times, and participant preferences were recorded for comparison.

The experimental results show that the overall usage process of the age-friendly smart home prototype designed in this paper is smooth for the elderly in the operation test, which is basically consistent with the results of previous studies. However, there are shortcomings in the operation details. It is recommended to adjust the main function bar to the lower part of the home page, reduce the interaction steps for users to use the scene functions, use color and switch button function to indicate the switching status of the device, add text labels for the buttons, use plus icons and text logos, etc.. It makes the meaning clearer, reduces the user's cognitive load, and helps the user understand and find. It shows that the 18 mm function buttons, the up-and-down sliding layout and the minimalist style received the best subjective evaluation from seniors. In terms of color, green is recommended as the theme color, with highlighted green and gray highlighting to guide seniors to perform correct tapping operations.

The results of these studies improve the operability and aesthetic comfort of smart home interaction prototyping in age-friendly scenarios, both functionally and visually, and enhance the ease of use of the interface to provide a better experience for the elderly when using smart homes.

## Data availability statement

The raw data supporting the conclusions of this article will be made available by the authors, without undue reservation.

## Ethics statement

The studies involving human participants were reviewed and approved by Nanjing Forestry University. The patients/participants provided their written informed consent to participate in this study.

## Author contributions

CZ contributed framework ideas, experimental design, and analysis methods. WZ conducted the experimental manipulations, collection, and analysis of primary data, and plotted the graphs. TH and HZ assisted in acquiring and analyzing the data for the work and assisted in graphing the figures. JK guided the refinement of the language, analyzed the data, critically revised important intellectual content and the layout of the article, and ensured that issues related to the accuracy or completeness of any portion of the work were properly investigated and resolved. All authors contributed to the article and approved the submitted version.
